# Repurposing FDA approved drugs as radiosensitizers for treating hypoxic prostate cancer

**DOI:** 10.1186/s12894-021-00856-x

**Published:** 2021-07-01

**Authors:** Becky A. S. Bibby, Niluja Thiruthaneeswaran, Lingjian Yang, Ronnie R. Pereira, Elisabet More, Darragh G. McArt, Paul O’Reilly, Robert G. Bristow, Kaye J. Williams, Ananya Choudhury, Catharine M. L. West

**Affiliations:** 1 Translational Radiobiology Group, Division of Cancer Science, School of Medical Sciences, Faculty of Biology, Medicine and Health, University of Manchester, Manchester Academic Health Sciences Centre, The Christie NHS Foundation Trust, Wilmslow Road, Manchester, M20 4BX UK; 2 Sydney Medical School, University of Sydney, Camperdown, Australia; 3 Translational Oncogenomics, CRUK Manchester Institute and CRUK Manchester Centre, Manchester, UK; 4 Centre for Cancer Research and Cell Biology, Queen’s University Belfast, Belfast, UK; 5 School of Pharmacy and Pharmaceutical Sciences, University of Manchester, Manchester, UK

## Abstract

**Background:**

The presence of hypoxia is a poor prognostic factor in prostate cancer and the hypoxic tumor microenvironment promotes radioresistance. There is potential for drug radiotherapy combinations to improve the therapeutic ratio. We aimed to investigate whether hypoxia-associated genes could be used to identify FDA approved drugs for repurposing for the treatment of hypoxic prostate cancer.

**Methods:**

Hypoxia associated genes were identified and used in the connectivity mapping software QUADrATIC to identify FDA approved drugs as candidates for repurposing. Drugs identified were tested in vitro in prostate cancer cell lines (DU145, PC3, LNCAP). Cytotoxicity was investigated using the sulforhodamine B assay and radiosensitization using a clonogenic assay in normoxia and hypoxia.

**Results:**

Menadione and gemcitabine had similar cytotoxicity in normoxia and hypoxia in all three cell lines. In DU145 cells, the radiation sensitizer enhancement ratio (SER) of menadione was 1.02 in normoxia and 1.15 in hypoxia. The SER of gemcitabine was 1.27 in normoxia and 1.09 in hypoxia. No radiosensitization was seen in PC3 cells.

**Conclusion:**

Connectivity mapping can identify FDA approved drugs for potential repurposing that are linked to a radiobiologically relevant phenotype. Gemcitabine and menadione could be further investigated as potential radiosensitizers in prostate cancer.

**Supplementary Information:**

The online version contains supplementary material available at 10.1186/s12894-021-00856-x.

## Background

The goal of drug repurposing is to find new clinical indications for existing pharmaceuticals that are currently on the market or failed in phase II/III trials. Repurposing is feasible because disease mechanisms are multifactorial and small drug molecules have multiple targets. Drug repurposing is both time and cost effective since the pharmacology and toxicity profile of approved drugs are already established. Approximately 30% of food and drug authority (FDA) applications for repurposed drugs are approved compared with 10% for new drugs [1, 2]. There is also potential for drug radiotherapy combinations to improve therapeutic ratios and enhance efficacy without increasing toxicity [3].

Drug and transcriptomic connectivity mapping can identify drug candidates for repurposing. The most widely used method is CMap (connectivity map project) which connects gene expression profiles to drugs based on data obtained from human cell lines treated with FDA approved drugs [4]. CMap currently has over one million gene expression profiles from multiple cell lines treated with approximately 20,000 compounds. Since the release of the data sets from the library of integrated cellular signatures (LINCS) program, additional connectivity mapping algorithms have been developed such as the Queens University Belfast Accelerated Drug and Transcriptomic Connectivity (QUADrATiC) program [5]. This software provides an improved and rapid method for calculating connection scores between the LINCS database and FDA approved compounds in order to identify drugs with the potential to reverse the biology or phenotype associated with the genes of interest [6]. Any positive hit from this algorithm has already been identified as a safe therapeutic and can be progressed into a Phase I/II radiotherapy combination trial.

Computation-based approaches to drug repurposing provide an opportunity to identify novel agents to combine with radiotherapy [7]. Prostate cancer is the most common malignancy in men with just under 50,000 new cases diagnosed in the UK and 450,000 in Europe each year [8–10]. The local disease is managed with combinations of surgery, radiotherapy and hormones. The presence of hypoxia increases treatment resistance in prostate cancer patients treated with surgery or radiotherapy [11]. Targeting hypoxia in combination with radiotherapy has not been widely studied in prostate cancer, however, two single arm trials suggest the approach is feasible [12, 13]. To date the most extensive and convincing evidence for hypoxia modification in combination with radiotherapy comes from studies in head and neck cancer and muscle-invasive bladder cancer [14–16]. The retrospective analysis of hypoxia gene signature biomarkers within clinical trials confirmed patients with hypoxic tumors benefit most from hypoxia modification [14, 17]. Hypoxia gene signature biomarkers have been derived for multiple cancers and do not necessarily recapitulate across disease sites hence disease site-specific signatures have been developed [18]. Recently, we derived a gene signature for assessing hypoxia in prostate cancer [19]. The aim of this study was to investigate whether the transcription network associated with our hypoxia gene signature could be used in QUADrATIC to identify FDA approved drugs for potential repurposing for the treatment of hypoxic prostate cancer.

## Methods

### Identifying hypoxia associated genes

Genes significantly differentially expressed after 24 h exposure to 1% oxygen in more than two cell lines (DU145, PC3, LNCaP, PNT2) were identified previously as seed genes. For each cell line, genes differentially expressed between normoxia and hypoxia conditions across triplicates were selected using a rank product probability of false positive rate < 0.05 [19]. The 848 seed genes were used to build gene co-expression networks using the publicly available GSE21032 or TCGA cohorts [18, 20]. Gene co-expression networks were assembled and partitioned into gene modules using the Louvain method [19, 21]. Each module was tested for enrichment of the hypoxia seed genes using the Chi-square test. The upregulated genes from the gene module(s) significantly associated with a high percentage of hypoxia seed genes (FDR < 0.01) were used as input for QUADrATiC.

### STRING

The STRING database is a free online tool for exploring protein–protein interactions [22]. The user inputs their genes of interest, in this case the 103 GSE21032 and 66 TCGA hypoxia-associated genes, and the STRING database assembles a network of protein–protein interactions based on experimental evidence and predicted function. The associations in STRING include direct physical interactions and predicted indirect functional interactions. The protein–protein interactions are presented as networks, in which nodes represent proteins and the lines associations between proteins. The protein–protein association strength takes the form of a p-value that evaluates multiple channels of evidence as well as the chance of a random interaction between the protein pair [23].

### Connectivity mapping

QUADrATiC uses the LINCS database to identify connections between gene expression profiles and FDA-approved drugs [5]. The data in the LINCS database are compiled from in vitro cell line experiments. Two separate gene lists were used in the QUADrATiC software generated from GSE21032 (103 genes; Additional file 1: Table S2) and TCGA (66 genes; Additional file 1: Table S1). Genes were mapped to their corresponding probe(s) in the Affymetrix HG-133UA-na36 annotation (Additional file 1: Tables S3 and S4) and entered into the QUADrATIC software according to the most concordant drug response [24]. In other words, up-regulated genes associated with a poor prognosis (hazard ratio [HR] > 1) comprise the up-regulated set of the signature while up-regulated genes associated with a good prognosis (HR < 1) form the down-regulated set.

### Cell lines

Three prostate cancer cell lines were used to test drugs in vitro: DU145 (HTB-81), PC3 (CRL-1435) and LNCaP (CRL-1740). The cell lines were obtained from the American Type Culture Collection and authenticated by short tandem repeat profiling using the Promega Powerplex 21 system. Cell lines were cultured under normal conditions (37˚C, 5% CO_2_ in air), screened for mycoplasma and authenticated using short tandem repeat profiling. DU145 and LNCaP cells were cultured in RPMI 1640 (Sigma-Aldrich, UK) with 10% fetal bovine serum (FBS) and 2 mM L-glutamine (Sigma-Aldrich, UK). PC3 cells were cultured in Ham's F12 (Gibco, ThermoFisher, UK) with 10% FBS and 2 mM L-glutamine (Sigma-Aldrich, UK).

### Drug preparation

Menadione (Selleck Chem, Texas, USA), gemcitabine (Selleck Chem, Texas, USA) and tirapazamine (APExBIO, Texas, USA) were purchased in lyophilized form. Drugs were reconstituted to a concentration of 50 mM in dimethyl sulfoxide (DMSO) as recommended by the manufacturer. Stock solutions were aliquoted and stored at -80 °C; aliquots were not repeatedly freeze thawed.

### Sulforhodamine B (SRB) assays

Cells were seeded into 96 well plates and allowed to adhere overnight in the incubator. The next day plates were treated with 0, 2, 5, 10, 50 or 100 µM of drug or DMSO vehicle control. Following application of the drug, cells were immediately moved into a 0.1% O_2_ hypoxia chamber (Don Whitley Scientific, Bingley, UK) or kept in a normoxia incubator for 24 h. The assay endpoint was either 24 h or 4 days post-treatment. For the 24 h time point, the cells were fixed and stained after 24 h incubation with the drug. For the 4-day time point, the drug was removed after 24 h and fresh media added to the cells. Plates were incubated for a further 4 days in normoxia. Cells were fixed and then stained with sulforhodamine B according to the published method [25].

### Clonogenic assays

Cells were seeded into plug seal T25 flasks and allowed to adhere overnight in the incubator, with plug seal caps kept loose. The next day media were removed and replaced with fresh media containing the drug (10 µM menadione or 8 nM gemcitabine) or DMSO (vehicle control). Untreated control flasks had fresh media applied. Cells exposed to normoxia, with or without the drug, were incubated with plug seal caps left loose in the incubator for 24 h. Cells exposed to 0.1% oxygen for 24 h, with or without the drug, were incubated with plug seal caps left loose in the H35 hypoxystation (Don Whitley Scientific, Bingley, UK). After 24 h plug seal caps were tightened on the flasks in the hypoxystation to maintain hypoxic conditions during irradiation. Cells were irradiated with x-rays delivered at 0.95 Gy/min using a Faxitron X-ray machine (Tucson, Arizona, USA). After irradiation cells were immediately harvested, counted and seeded onto 6-well plates [26]. Plates were incubated for 7–21 days until colonies had formed, fixed and stained using crystal violet solution (70% methanol (v/v), 0.1% crystal violet). Plates were imaged on a GelCount machine (Oxford Optronix, Abingdon, UK) and colonies counted using an optimized CHARM algorithm in the GelCount software. The surviving fraction was calculated for each biological repeat (experiments ran on separate days with cells of a different passage) using the mean number of colonies across the six individual wells. The concentration of DMSO was 0.02% (v/v) for menadione and 0.000016% (v/v) for gemcitabine. Sensitizer enhancement ratio (SER) was calculated as the ratio between the doses needed for 1 log kill with or without the drug.

### Statistical analysis

Data are presented as mean ± standard error of the mean (SEM). GraphPad PRISM 8 was used to plot graphs, radiation survival curves were fitted with the linear quadratic model. Surviving fractions at 2 Gy (SF2) and SER values were extrapolated from the fit of the linear quadratic model using PRISM 8. GraphPad PRISM 8 was used to perform the F-test and statistical analysis, *p* values < 0.05 were considered significant.

## Results

### Hypoxia associated genes

**Fig. 1:**
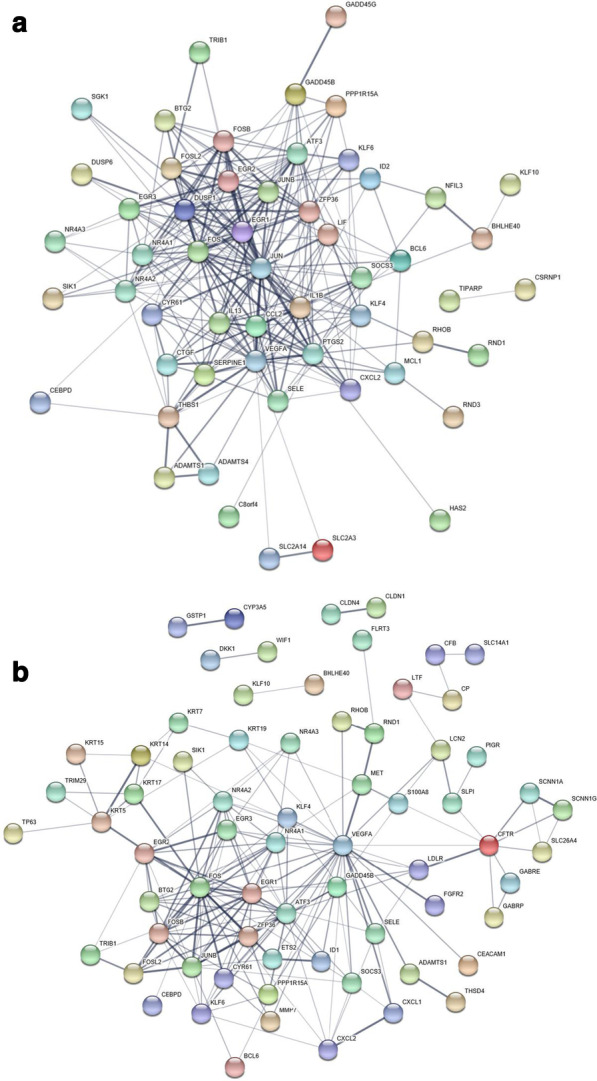
Prostate cancer hypoxia-associated genes interact at the protein level. The networks, generated using the STRING database, summarize predicted associations between proteins. The nodes represent proteins and only nodes with at least one connection are shown. The edges represent protein–protein interactions and the confidence of the interaction is indicated by the thickness of the edge. **a** Sixty-nine of the 103 hypoxia-associated genes identified in GSE21032 encoded proteins that interacted with at least one other protein in the network. On average each node had 3.6 connections and there are 185 edges representing protein–protein interactions. The observed number of edges in this network was more than expected at random with a protein–protein interaction enrichment value of *p* < 1 × 10^–16^, indicating a highly interconnected network of proteins. **b** Fifty-five of the 66 hypoxia-associated genes from the TCGA interacted with at least one other protein in the network and had an average 8.2 connections. There are 271 edges representing protein–protein interactions and number is more than expected at random (*p* < 1 × 10^–16^), indicating a highly interconnected network of proteins

Co-expression networks in the GSE21032 and TCGA cohorts identified hypoxia-associated genes (Additional file 1: Tables S1 and S2). Proteins encoded by the genes in the two networks interacted significantly (*p* < 1 × 10^–16^). Figure 1 illustrates the high level of connections between the proteins in the networks. The network plots show only proteins with at least one connection. Sixty-nine of the 103 genes (67%) in the GSE21032 list had at least one connection and there was an average of 3.6 connections per node. The TCGA gene list produced a more highly connected network of protein–protein interactions with 55 of the 66 genes (83%) having at least one connection and an average of 8.2 connections. The gene lists were applied independently to the QUADrATiC connectivity mapping software to identify FDA-approved drugs.

### QUADrATiC connectivity mapping

Drugs with negative connections were considered able to target the phenotype of interest, in this case hypoxia in prostate cancer. Using the GSE21032 hypoxia-associated genes there were 5,348 drugs with negative z-scores, of which 2,405 were nominally significant (*p* < 0.05). Using the TCGA hypoxia-associated genes there were 4,827 drugs with negative z-scores, of which 2,270 were nominally significant (*p* < 0.05). The QUADrATiC interface provided summary visualizations of the top connections as bubble charts and drug and cell line connections (Additional files 2, 3: Figure S1 and S2).

### Drug selection

The top 10 drugs from each of the two gene lists were ranked based on Z-score (Table 1). There were two prostate cell lines, PC3 and VCaP, in the connectivity mapping analysis but drug selection was not restricted to the highest ranking drugs identified in these cell lines (Table 2). The strength of the connection between the genes and the drug was considered more important than the cell line in which it was identified because (i) there were only two prostate cancer cell lines in the program and (ii) the importance of hypoxia across solid tumours. Two candidate drugs were selected for in vitro validation, menadione and gemcitabine, because they ranked in the top 10 in the GSE21032 and TCGA datasets in all (Table 1) and the prostate cancer (Table 2) cell lines. Menadione appeared twice in the top 10 ranked drugs in the GSE21032 and TCGA (Table 1) and was the only drug with a strong connection to the VCaP cell line in both datasets (Table 2). Gemcitabine appeared twice in the top 10 ranked drugs in the GSE21032 and once in the TCGA (Table 1) and had a strong connection to the PC3 cell line in both datasets (Table 2). Menadione and gemcitabine also had negative Z-scores in the PC3 and VCaP cell lines (Additional file 1: Table S5). The approved application, mechanism of action and reported peak plasma concentrations for menadione and gemcitabine are summarized in Table 3.

**Table 1:** Top 10 FDA-approved drugs identified using connectivity mapping

Ranking	GSE21032			TCGA		
	Drug	Cell line	Z-score^¶^	Drug	Cell line	Z-score^¶^
1	**MENADIONE**	HEPG2	– 15.8	CLADRIBINE	A375	– 17.5
2	**MENADIONE**	A375	– 14.0	**MENADIONE**	A375	– 15.6
3	**GEMCITABINE**	A375	– 13.1	HOMOHARRINGTONINE	PC3	– 15.0
4	NICLOSAMIDE	HEPG2	– 13.0	**MENADIONE**	HEPG2	– 14.6
5	**GEMCITABINE**	HCC515	– 12.1	NICLOSAMIDE	HEPG2	– 14.6
6	CLADRIBINE	A375	– 11.7	DIGITOXIN	A549	– 14.3
7	DIGITOXIN	A549	– 11.4	AZACITIDINE	A375	– 14.0
8	DIGOXIN	PC3	– 11.3	CLOFARABINE	A375	– 13.9
9	PENTAMIDINE	HEPG2	– 11.1	**GEMCITABINE**	HCC515	– 13.5
10	TENIPOSIDE	A375	– 11.1	CLADRIBINE	PC3	– 13.3

^¶^*p *values < 0.001

**Table 2:** Top 10 FDA-approved drugs identified using connectivity mapping in the prostate cancer cell lines

Ranking	GSE21032			TCGA		
	Drug	Cell line	Z-score	Drug	Cell line	Z-score
1	DIGOXIN	PC3	– 11.31	HOMOHARRINGTONINE	PC3	– 14.98
2	OUABAIN	PC3	– 10.48	CLADRIBINE	PC3	– 13.29
3	DIGITOXIN	PC3	– 9.83	DIGITOXIN	PC3	– 13.15
4	BISACODYL	PC3	– 9.56	**MENADIONE**	VCAP	– 12.56
5	CLOFARABINE	PC3	– 9.42	DIGOXIN	PC3	– 12.10
6	HOMOHARRINGTONINE	PC3	– 8.86	OUABAIN	PC3	– 11.59
7	ITRACONAZOLE	PC3	– 8.84	**GEMCITABINE**	PC3	– 11.56
8	**MENADIONE**	VCAP	– 8.69	AZACITIDINE	PC3	– 10.16
9	**GEMCITABINE**	PC3	– 8.36	BORTEZOMIB	VCAP	– 10.15
10	TENIPOSIDE	PC3	– 8.35	CEFACLOR	PC3	– 9.92

**Table 3:** Application, mechanism and peak plasma concentrations for menadione and gemcitabine

	Menadione	Gemcitabine
Approved application	Used in vitamin K deficiency and severe hypoprothrombinemia	Treatment of locally advanced or metastatic cancer
Mechanism of action	Synthetic vitamin K3. It is also an inhibitor of Siah2 (E3 ubiquitin ligase) ligase activity	Inhibition of DNA synthesis and inhibition of enzymes related to deoxynucleotide metabolism
Peak plasma concentration and equivalent in vitro dose	115–407 ng/mL^¶^ 668.6 ng–2.3 µM	3–6 µg/mL 11.2–22.4 µM
Equivalent in vitro dose	668.6 ng–2.3 µM	11.2–22.4 µM

^¶^Reported values for phytomenadione, doses up to 200 mg menadione are tolerated in humans

### Hypoxia selective cytotoxicity

**Fig. 2:**
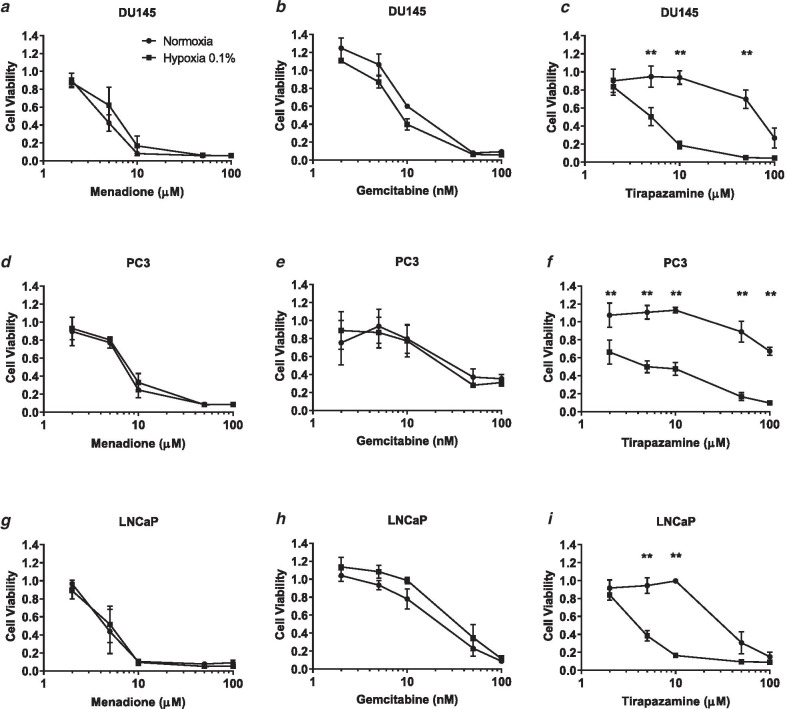
No hypoxia-selective cytotoxicity of menadione and gemcitabine 4 days post-treatment. DU145 (**a**–**c**), PC3 (D-F) and LNCaP (**g**–**i**) cell lines were exposed to menadione, gemcitabine or tirapazamine under normoxia or 0.1% O_2_ hypoxia. Only tirapazamine demonstrated hypoxia selective cytotoxicity in the cell lines. The data points represent the mean ± SEM of 2–4 values taken from each biological repeat, within each biological repeat there were 6 intra-assay replicates. Statistical analysis was performed using multiple t-tests with Holm-Sidak correction. **indicates *p* < 0.01

Menadione and gemcitabine were tested alongside the known bioreductively activated tirapazamine. In the DU145, PC3 and LNCaP cell lines tirapazamine demonstrated hypoxia selective cytotoxicity 24 h (Additional file 4: Figure S3) and 4 days (Fig. 2) following drug exposure). There was no loss in cytotoxicity for menadione and gemcitabine 4 days post-treatment in any of the three cell lines. However, hypoxic PC3 cells were more sensitive to 5 µM menadione and 10 µM gemcitabine than the normoxic cells after 24 h drug exposure (Additional file 4: Figure S3).

### Radiosensitization

**Fig. 3:**
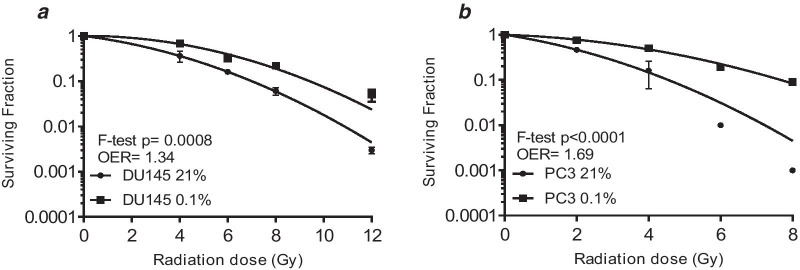
Hypoxia increased radioresistance in the DU145 and PC3 cell lines. Cell survival curves for DU145 (**a**) and PC3 (**b**) cells, irradiated under normoxia or 0.1% O_2_. The data points represent the mean ± SEM of 2–4 values taken from each biological repeat. OERs were calculated at SF10% and statistical analysis was performed using the F-test

**Fig. 4:**
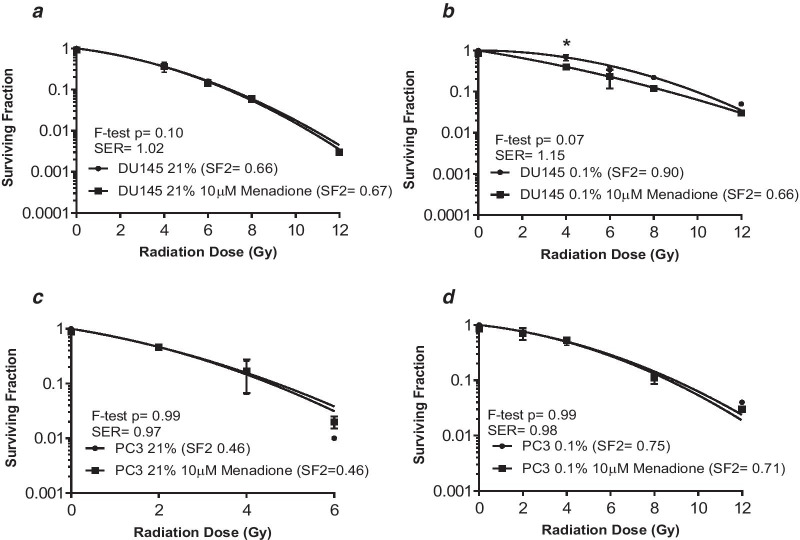
Menadione enhanced radiation response under hypoxic conditions in DU145 cells. **a** Menadione (10 µM, 24 h) did not enhance the radiosensitivity of normoxic DU145 cells. **b** The SER of hypoxic DU145 cells treated with menadione was 1.15 and at 4 Gy menadione significantly reduced surviving fraction compared to radiation alone. **c** In the PC3 cell line, menadione did not enhance the radiosensitivity of normoxic or **d** hypoxic cells. The data points represent the mean ± SEM of 2–4 values taken from each biological repeat, within each biological repeat there were 6 intra-assay replicates. Statistical analysis was performed using a paired t-test (ns = not significant, * indicates *p* < 0.05). SERs were calculated at SF10% and statistical analysis was performed using the F-test

**Fig. 5:**
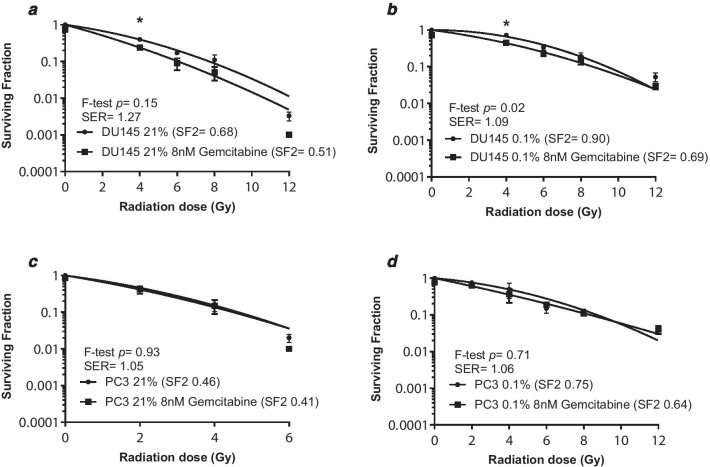
Gemcitabine enhanced radiation response under normoxic and hypoxic conditions in the DU145 cells. **a** Gemcitabine (8 nM, 24 h) enhanced the radiosensitivity of normoxic DU145 cells, the SER was 1.27 and at 4 Gy gemcitabine significantly reduced surviving fraction compared to radiation alone. **b** Under hypoxia the SER of gemcitabine was 1.09 and at 4 Gy gemcitabine significantly reduced surviving fraction compared to radiation alone. **c** In the PC3 cell line, gemcitabine did not enhance the radiosensitivity of normoxic or **d** hypoxic cells. The data points represent the mean ± SEM of 2–4 values taken from each biological repeat, within each biological repeat there were 6 intra-assay replicates. Statistical analysis was performed using a paired t-test (ns = not significant, * indicates *p* < 0.05). SERs were calculated at SF10% and statistical analysis was performed using the F-test

The radiosensitizing ability of the two FDA approved drugs was studied in DU145 and PC3 cells. The rationale for selecting DU145 in addition to PC3 for the radiosensitization experiments is because this cell line was derived from human tissue, whereas the LNCaP cell line was initially derived from human tissue but cultured in a mouse model.Vehicle control experiments confirmed DMSO did not alter surviving fraction compared to the untreated controls and DMSO plus radiation did not alter surviving fraction compared to radiation alone (Additional file 5: Figure S4). Figure 3 shows survival curves for the cells irradiated in normoxia and hypoxia. Oxygen enhancement ratio (OERs) calculated at the 10% survival level were 1.34 for DU145 and 1.69 for PC3 cells. Figure 4 shows menadione was a weak radiosensitizer in DU145 cells only. The SER for menadione in DU145 cells was 1.02 in normoxia and 1.15 in hypoxia. Figure 5 shows gemcitabine was also a weak radiosensitizer in DU145 cells only. The SER for gemcitabine was 1.27 in normoxia and 1.09 in hypoxia.

## Discussion

Our study demonstrated how a gene expression network associated with hypoxia can be used in the QUADrATIC software to identify FDA approved drugs with the potential to be repurposed. The candidate drugs selected for in vitro validation, menadione and gemcitabine, showed similar cytotoxicity in normoxia and hypoxia in three cell lines. There was also evidence that the drugs were weak radiosensitizers in normoxia and hypoxia in one of the cell lines studied.

Although menadione and gemcitabine did not demonstrate hypoxia-selective cytotoxicity the drugs had similar efficacy in normoxia and hypoxia. In contrast, many chemotherapeutic agents (e.g., cisplatin, 5-FU and doxorubicin) have reduced cytotoxicity in vitro in hypoxia [27–30]. In general, few studies compared the cytotoxicity of chemotherapeutic agents in normoxia and hypoxia in prostate cancer cell lines. Docetaxel, a first line systemic treatment for prostate cancer, has been shown to have reduced cytotoxicity in hypoxia (1% and 0.1% oxygen) in a range of cell lines [27]. However, a study in DU145 and 22Rv1 cells showed similar docetaxel cytotoxicity in normoxia and hypoxia (0.5% oxygen) [31]. It is uncommon for drugs studied in radiotherapy combination trials to have had their efficacy first tested under hypoxia. In future, pre-clinical testing as a justification for trial design should involve in vitro testing in hypoxia as well as normoxia.

As expected, we have shown the hypoxia selective toxicity of tirapazamine. Although previously studied in PC3 cells, this is the first study of tirapazamine in DU145 and LNCaP cells. The IC_50_ for PC3 was 5 μM (4 days post-treatment after 24 h at 0.1% O_2_), which compares with literature reported IC_50_ doses for tirapazamine in PC3 cells of 15 µM [4 h anoxia] and 22 µM (48 h at 3% O_2_) [32, 33]. Tirapazamine has been shown to enhance the effect of castration induced hypoxia by inducing apoptosis and subsequently reducing tumor volume [34]. Furthermore, hypoxia induces adaptive androgen independence and confers resistance to androgen deprivation therapy (ADT) [35]. Tirapazamine in combination with ADT has the potential to eliminate hypoxic tumor cells and prevent the development of ADT resistant clones. However, a Phase III trial that randomised head and neck cancer patients to chemoradiotherapy alone or with tirapazamine showed no benefit [36].

To identify gene expression changes in response to hypoxia the cells were exposed to 1% O_2_ because the HIF-1 transcription factor is stabilised and changes in gene expression occurs. However, the level of O_2_ at which significant resistance to radiation is observed is < 0.13% hence the in vitro experiments were performed at 0.1% O_2_ [37]. The physiological level of oxygen in the normal prostate is 3.4–3.9% but oxygen levels in prostate tumor tissue are in the range of 0.3–1.2% [38]. Normoxia in this study is 21% O_2_ which is supraphysiological compared to the level of oxygen in the prostate gland. However, in vitro cell lines are routinely established and cultured in the laboratory under these conditions and have adapted to grow at 21% O_2_ and therefore the difference in gene expression may not reflect in vivo changes.

The OERs for the DU145 and PC3 cell lines exposed to 0.1% O_2_ for 24 h were low over the dose ranges studied but similar to those previously reported for DU145 and PC3 with the exception of PC3 transfected with mir210 inhibitors giving an OER of ~ 2 [39–42]. Furthermore, in vitro studies have reported varying OERs depending on the duration under hypoxia with OER decreasing with time of exposure under hypoxia for the same cell lines [41].

The guidelines for preclinical and early phase assessment of radiosensitizers state that relatively low SER values in the range 1.2–1.5 may indicate a useful effect, particularly if sensitization occurs at clinically relevant doses of radiation [43]. In the DU145 cell line, the SER for menadione under hypoxia was 1.15 which is comparable to the SER of nimorazole in the head and neck cancer cell lines FaDu (SER 1.14) and UMSCC47 (SER 1.13) [44]. The SER of 1.27 for gemcitabine in normoxia was comparable to previously reported values in the range of 1.1–3 [45]. This normoxic radiosensitization with gemcitabine is comparable to the radiosensitizing effects of other chemotherapeutic agents such as 5-FU [46].

Menadione, also known as vitamin K_3_, is a quinone and synthetic vitamin that can be converted into active vitamin K_2_ in the body. Menadione induces the production of reactive oxygen species (ROS) through redox cycling and disrupts the interaction between HIF-1a and its coactivator p300 thus inhibiting HIF-1a transcriptional activity [47–49]. Apatone (menadione and vitamin C) has shown prostate cancer antitumor activity in vitro and the toxicity profile was favorable in a phase I/IIa study [50, 51]. PSA velocity and PSA doubling time decreased in 15 of 17 patients suggesting value in progressing apatone into the Phase III setting.

Gemcitabine is a chemotherapeutic agent used to treat several cancers, it is a nucleoside analogue that is incorporated into the DNA and inhibits DNA synthesis resulting in cell death. Nucleoside analogues such as gemcitabine are considered as potential radiosensitizers because they inhibit the repair of radiation induced DNA damage [52]. In muscle invasive bladder cancer evidence from phase I/II trials supports the concurrent administration of gemcitabine and radiotherapy as a bladder preservation strategy [53, 54]. In the breast cancer cell line MDA-MB-231, the SER for gemcitabine and radiation under hypoxia was 1.59 and under normoxia 1.70 [55]. The radiosensitizing effects of gemcitabine in breast cancer are greater than the effects reported in this study for prostate cancer. However, the radiosensitizing effect of gemcitabine is greater under normoxia agrees with our findings.

A limitation of our study is that radiosensitizing effects were weak and only observed in DU145 cells. This cell line is derived from a central nervous system metastasis of primary prostate adenocarcinoma [56]. In comparison, the PC3 cell line is characteristic of neuroendocrine-like prostate cancer, which represents less than 2% of cases and is biologically distinct from the more common adenocarcinoma subtype [57, 58]. Both patients had prior treatment with hormonal therapy before cell lines were derived. Nonetheless, they are the most commonly used prostate cancer cell lines. Interestingly, a study investigating the radiosensitizing effect of vorinostat in prostate cell lines reported a radiosensitizing effect under normoxia and hypoxia in the DU145 cells but no effect in PC3 cells [59]. A second limitation is that effects were only studied in cell lines grown as monolayers, an in vitro spheroid model may incorporate physiological hypoxia into the model. Although it is worth noting that, the in vitro data utilized by the QUADrATIC connectivity mapping software was obtained from monolayer cultured under 21% O_2_.

Two approaches have been employed to identify FDA-approved drug for repurposing: in silico analytics and experimental screening studies [2]. In a high-throughput oxygen consumption screen, atovaquone was shown to reduce oxygen consumption [60]. Atovaquone is an FDA approved anti-malarial with a similar chemical structure to menadione. Atovaquone was shown to reduce tumor hypoxia and increase radiosensitivity at pharmacological concentrations in spheroids in vitro and in vivo [60]. Interestingly, the drug did not alter the radiosensitivities of hypoxic cells grown as monolayers suggesting that atovaquone affects the tumor microenvironment rather than increasing the intrinsic radiosensitivity of cells [60]. Atovaquone is currently being tested in phase I clinical trial for its ability to alleviate tumor hypoxia in lung cancer (NCT02628080).

## Conclusion

In summary, this study highlights how connectivity mapping can be used to identify FDA approved drugs linked to biological phenotypes for potential repurposing. Our work shows the importance of downstream validation and proof-of-principle studies when identifying drugs for repurposing using in silico analytical approaches. Tirapazamine is an effective hypoxia-selective agent in prostate cancer cell lines and could be tested alongside ADT in future studies. In the DU145 cell line, menadione was a hypoxic radiosentiziter and gemcitabine was a normoxic radiosensitizer. Gemcitabine could be further investigated given that the guidelines for preclinical and early phase assessment of radiosensitizers report an SER in the range of 1.2–1.5 could be clinically useful.

## Supplementary Information

**Additional file 1.** **Supplementary Table 1.** GSE21032 hypoxia associated genes n = 103. **Supplementary Table 2.** TCGA hypoxia associated genes n = 66. **Supplementary Table 3.** GSE21032 hypoxia associated genes Affymetrix probe IDs. **Supplementary Table 4.** TCGA hypoxia associated genes Affymetrix probe IDs. **Supplementary Table 5.** The Z-scores for menadione and gemcitabine in the prostate cancer cell lines.

**Additional file 2.** **Supplementary Figure 1.** Bubble plots representing the strength of the top negative connections identified by QUADrATiC. The larger the bubble the stronger the connection between the identified drug and the input genes. The drug and the cell line, that the connection was derived from, are shown in the bubbles. (**A**) GSE21032 (**B**) TCGA.

**Additional file 3.** **Supplementary Figure 2.** Top drug and cell line connections identified by QUADrATiC. The plots represent the top ranked drugs and the cell line that the connection was derived from in the LINCs database. (**A**) GSE21032 (**B**) TCGA.

**Additional file 4.** **Supplementary Figure 3.** No loss of cytotoxicity of menadione and gemcitabine in hypoxia versus normoxia after 24 h. DU145 (**A**–**C**), PC3 (**D**–**F**) and LNCaP (**G**–**I**) cell lines were exposed to menadione, gemcitabine or tirapazamine under normoxia or 0.1% O2 hypoxia. Three independent experiments were carried out, with six intra-assay replicates per experiment. Data points represent the mean ± SEM, statistical analysis was performed using a t-test with Holm-Sidak correction; **p < 0.01).

**Additional file 5.** **Supplementary Figure 4.** (**A**) Under normoxia DMSO at a concentration of 0.02% (v/v) did not alter the surviving fraction of DU145 cells that were mockirradiated or irradiated with 4 Gy. (**B**) Under hypoxia DMSO at a concentration of 0.02% (v/v) did not alter the surviving fraction of DU145 cells that were mock irradiated or irradiated with 4 Gy. Data points represent the mean ± SEM of 3 biological repeats.

## Data Availability

All data analysed during this study are included in this published article and its supplementary information files. Generated data from each experimental repeat are available from the corresponding author on reasonable request.

## Abbreviations

CMap: Connectivity map project
LINCS: Library of integrated cellular signatures
QUADrATiC: Queens University Belfast Accelerated Drug and Transcriptomic Connectivity
TCGA: The Cancer Genome Atlas
OER: Oxygen enhancement ratio
SER: Sensitizer enhancement ratio
