# Supplementary material for: Repurposing FDA approved drugs as radiosensitizers for treating hypoxic prostate cancer
Source: BMC Urol. 2021 Jul 1;21:96. doi: 10.1186/s12894-021-00856-x (PMC8247203; doi:10.1186/s12894-021-00856-x)
Supplement: Supplementary file 1 — Additional file 1. Supplementary Table 1. GSE21032 hypoxia associated genes n = 103. Supplementary Table 2. TCGA hypoxia associated genes n = 66. Supplementary Table 3. GSE21032 hypoxia associated genes Affymetrix probe IDs. Supplementary Table 4. TCGA hypoxia associated genes Affymetrix probe IDs. Supplementary Table 5. The Z-scores for menadione and gemcitabine in the prostate cancer cell lines. [file 12894_2021_856_MOESM1_ESM.docx]

**Supplementary Table 1.** GSE21032 hypoxia associated genes n=103.

| Up-regulated Genes Good Prognosis (HR<1; Quadratic ‘Down’ genes) | | | | | | | | | | |
| --- | --- | --- | --- | --- | --- | --- | --- | --- | --- | --- |
| *ADAMTS1* | *LXN* | | *JUNB* | | *OLFM4* | | *SCUBE2* | | *SCNN1A* | |
| *PDLIM1* | *MMP26* | | *NR4A1* | | *SELE* | | *TNS4* | | *LDLR* | |
| *CRISPLD2* | *CPA6* | | *NR4A2* | | *TP63* | | *TFCP2L1* | | *CXCL2* | |
| *ETS2* | *CYR61* | | *SIK1* | | *KRT15* | | *KRT14* | | *GABRP* | |
| *FGFR2* | *SLC14A1* | | *SOCS3* | | *ID1* | | *KRT19* | | *PIGR* | |
| *GABRE* | *THSD4* | | *ZFP36* | | *CYP3A5* | | *CFB* | | *CLDN4* | |
| *KLF10* | *RHOB* | | *CEBPD* | | *KLF4* | | *SLC26A4* | | *CXCL1* | |
| *APOBEC3C* | *BTG2* | | *DKK1* | | *KRT5* | | *HES1* | | *LCN2* | |
| *BHLHE40* | *FOSL2* | | *RND1* | | *TRIM29* | | *EGR3* | | *CYP24A1* | |
| *MET* | *CSRNP1* | | *B3GNT5* | | *CFTR* | | *KLF6* | | *KRT7* | |
| *NR4A3* | *EGR1* | | *CEACAM1* | | *HOXD10* | | *PPP1R15A* | | *SCNN1G* | |
| *BCL6* | *EGR2* | | *ERRFI1* | | *KRT17* | | *RASD1* | | *SLC6A14* | |
| *DSC3* | *FOSB* | | *CLDN1* | | *KRT23* | | *TRIB1* | | *S100A8* | |
| *GSTP1* | *FOS* | | *CP* | | *LTF* | | *WEE1* | | *VEGFA* | |
| *ITGB6* | *GADD45B* | | *FLRT3* | | *MMP7* | | *C8orf4* | | *ATF3* | |
| *CD177* | *CYP4X1* | |  | |  | |  | |  | |
| Up-regulated Genes Poor Prognosis (HR>1; Quadratic ‘Up’ genes) | | | | | | | | | | |
| *PTPRT* | | *ZNF30* | | *SLC5A1* | | *SLPI* | | *CHI3L2* | | *CPB1* |
| *WIF1* | | *TGM4* | | *SERPINEB11* | | *CHL1* | | *NRG4* | |  |

**Supplementary Table 2.** TCGA hypoxia associated genes n=66.

| Up-regulated Genes Good Prognosis (HR<1; Quadratic ‘Down’ genes) | | | | | | | | | | |
| --- | --- | --- | --- | --- | --- | --- | --- | --- | --- | --- |
| *BTG2* | *KLF4* | | *TIPARP* | | *RASD1* | | *CSRNP1* | | *DNAJB4* | |
| *JUN* | *RHOB* | | *EGR1* | | *PPP1R15A* | | *DUSP1* | | *FOS* | |
| *ZFP36* | *EGR3* | | *NR4A1* | | *PTGS2* | | *ATF3* | | *CYR61* | |
| *FOSB* | *RND3* | | *MCL1* | | *APOLD1* | | *RND1* | | *JUNB* | |
| *SELE* | *GADD45B* | | *KLF6* | | *KLF10* | | *EGR2* | | *BCL6* | |
| *ADAMTS1* | *ID2* | | *ERRFI1* | | *NFIL3* | | *TSC22D1* | | *SOCS3* | |
| *CXCL2* | *SLC2A3* | | *SIK1* | | *NR4A3* | | *SLC10A6* | | *SLC2A14* | |
| *SGK1* | *GEM* | | *FOSL2* | | *NR4A2* | | *C8orf4* | | *IL1B* | |
| *CEBPD* | *CLDN4* | | *TRIB1* | | *CD200* | | *THBS1* | |  | |
| Up-regulated Genes Poor Prognosis (HR>1; Quadratic ‘Up’ genes) | | | | | | | | | | |
| *CTGF* | | *HAS2* | | *ADAMTS4* | | *BHLHE40* | | *LIF* | | *DUSP6* |
| *VEGFA* | | *CCL2* | | *SERPINE1* | | *PNP* | | *GADD45G* | | *IL13* |
| *CCNL1* | |  | |  | |  | |  | |  |

**Supplementary Table 3.** GSE21032 hypoxia associated genes Affymetrix probe IDs.

| Affymetrix Probe ID | Gene Symbol |
| --- | --- |
| 200824_at | GSTP1 |
| 201169_s_at | BHLHE40 |
| 201170_s_at | BHLHE40 |
| 201235_s_at | BTG2 |
| 201236_s_at | BTG2 |
| 201289_at | CYR61 |
| 201328_at | ETS2 |
| 201329_s_at | ETS2 |
| 201428_at | CLDN4 |
| 201473_at | JUNB |
| 201531_at | ZFP36 |
| 201650_at | KRT19 |
| 201693_s_at | EGR1 |
| 201694_s_at | EGR1 |
| 201820_at | KRT5 |
| 202014_at | PPP1R15A |
| 202018_s_at | LTF |
| 202067_s_at | LDLR |
| 202068_s_at | LDLR |
| 202241_at | TRIB1 |
| 202340_x_at | NR4A1 |
| 202357_s_at | CFB |
| 202393_s_at | KLF10 |
| 202504_at | TRIM29 |
| 202672_s_at | ATF3 |
| 202768_at | FOSB |
| 202917_s_at | S100A8 |
| 203140_at | BCL6 |
| 203393_at | HES1 |
| 203394_s_at | HES1 |
| 203395_s_at | HES1 |
| 203453_at | SCNN1A |
| 203510_at | MET |
| 203638_s_at | FGFR2 |
| 203639_s_at | FGFR2 |
| 203973_s_at | CEBPD |
| 204213_at | PIGR |
| 204259_at | MMP7 |
| 204470_at | CXCL1 |
| 204537_s_at | GABRE |
| 204602_at | DKK1 |
| 204621_s_at | NR4A2 |
| 204622_x_at | NR4A2 |
| 204734_at | KRT15 |
| 204846_at | CP |
| 205043_at | CFTR |
| 205044_at | GABRP |
| 205157_s_at | KRT17 |
| 205249_at | EGR2 |
| 205409_at | FOSL2 |
| 205765_at | CYP3A5 |
| 205856_at | SLC14A1 |
| 206032_at | DSC3 |
| 206033_s_at | DSC3 |
| 206115_at | EGR3 |
| 206211_at | SELE |
| 206359_at | SOCS3 |
| 206360_s_at | SOCS3 |
| 206504_at | CYP24A1 |
| 206529_x_at | SLC26A4 |
| 206576_s_at | CEACAM1 |
| 207295_at | SCNN1G |
| 207373_at | HOXD10 |
| 207382_at | TP63 |
| 207574_s_at | GADD45B |
| 207978_s_at | NR4A3 |
| 208078_s_at | SIK1 |
| 208083_s_at | ITGB6 |
| 208084_at | ITGB6 |
| 208225_at | FGFR2 |
| 208228_s_at | FGFR2 |
| 208229_at | FGFR2 |
| 208234_x_at | FGFR2 |
| 208690_s_at | PDLIM1 |
| 208937_s_at | ID1 |
| 208960_s_at | KLF6 |
| 208961_s_at | KLF6 |
| 209016_s_at | KRT7 |
| 209189_at | FOS |
| 209304_x_at | GADD45B |
| 209305_s_at | GADD45B |
| 209351_at | KRT14 |
| 209498_at | CEACAM1 |
| 209584_x_at | APOBEC3C |
| 209774_x_at | CXCL2 |
| 209863_s_at | TP63 |
| 209959_at | NR4A3 |
| 210056_at | RND1 |
| 210226_at | NR4A1 |
| 210512_s_at | VEGFA |
| 210513_s_at | VEGFA |
| 210610_at | CEACAM1 |
| 210764_s_at | CYR61 |
| 211001_at | TRIM29 |
| 211002_s_at | TRIM29 |
| 211143_x_at | NR4A1 |
| 211193_at | TP63 |
| 211194_s_at | TP63 |
| 211195_s_at | TP63 |
| 211398_at | FGFR2 |
| 211399_at | FGFR2 |
| 211400_at | FGFR2 |
| 211401_s_at | FGFR2 |
| 211527_x_at | VEGFA |
| 211599_x_at | MET |
| 211610_at | KLF6 |
| 211834_s_at | TP63 |
| 211883_x_at | CEACAM1 |
| 211889_x_at | CEACAM1 |
| 211920_at | CFB |
| 212099_at | RHOB |
| 212171_x_at | VEGFA |
| 212236_x_at | KRT17 |
| 212531_at | LCN2 |
| 212533_at | WEE1 |
| 212768_s_at | OLFM4 |
| 213006_at | CEBPD |
| 213560_at | GADD45B |
| 213807_x_at | MET |
| 213816_s_at | MET |
| 214105_at | SOCS3 |
| 214234_s_at | CYP3A5 |
| 214235_at | CYP3A5 |
| 214370_at | S100A8 |
| 215026_x_at | SCNN1A |
| 215702_s_at | CFTR |
| 215703_at | CFTR |
| 215711_s_at | WEE1 |
| 215990_s_at | BCL6 |
| 216248_s_at | NR4A2 |
| 216979_at | NR4A3 |
| 217005_at | LDLR |
| 217026_at | CFTR |
| 217103_at | LDLR |
| 217173_s_at | LDLR |
| 217264_s_at | SCNN1A |
| 218182_s_at | CLDN1 |
| 218541_s_at | C8orf4 |
| 218729_at | LXN |
| 218880_at | FOSL2 |
| 218881_s_at | FOSL2 |
| 218963_s_at | KRT23 |
| 219153_s_at | THSD4 |
| 219197_s_at | SCUBE2 |
| 219250_s_at | FLRT3 |
| 219669_at | CD177 |
| 219735_s_at | TFCP2L1 |
| 219795_at | SLC6A14 |
| 220266_s_at | KLF4 |
| 220541_at | MMP26 |
| 221541_at | CRISPLD2 |
| 221841_s_at | KLF4 |
| 222162_s_at | ADAMTS1 |
| 222265_at | TNS4 |
| 37028_at | PPP1R15A |
| 203021_at | SLPI |
| 204591_at | CHL1 |
| 204712_at | WIF1 |
| 205509_at | CPB1 |
| 205948_at | PTPRT |
| 206260_at | TGM4 |
| 206628_at | SLC5A1 |
| 213060_s_at | CHI3L2 |
| 217566_s_at | TGM4 |
| 217567_at | TGM4 |

**Supplementary Table 4.** TCGA hypoxia associated genes Affymetrix probe IDs.

| Affymetrix Probe ID | Gene Symbol |
| --- | --- |
| 200796_s_at | MCL1 |
| 200797_s_at | MCL1 |
| 200798_x_at | MCL1 |
| 201041_s_at | DUSP1 |
| 201044_x_at | DUSP1 |
| 201107_s_at | THBS1 |
| 201108_s_at | THBS1 |
| 201109_s_at | THBS1 |
| 201110_s_at | THBS1 |
| 201235_s_at | BTG2 |
| 201236_s_at | BTG2 |
| 201289_at | CYR61 |
| 201428_at | CLDN4 |
| 201464_x_at | JUN |
| 201465_s_at | JUN |
| 201466_s_at | JUN |
| 201473_at | JUNB |
| 201531_at | ZFP36 |
| 201565_s_at | ID2 |
| 201566_x_at | ID2 |
| 201693_s_at | EGR1 |
| 201694_s_at | EGR1 |
| 201739_at | SGK1 |
| 202014_at | PPP1R15A |
| 202241_at | TRIB1 |
| 202340_x_at | NR4A1 |
| 202393_s_at | KLF10 |
| 202497_x_at | SLC2A3 |
| 202498_s_at | SLC2A3 |
| 202499_s_at | SLC2A3 |
| 202672_s_at | ATF3 |
| 202768_at | FOSB |
| 203140_at | BCL6 |
| 203574_at | NFIL3 |
| 203810_at | DNAJB4 |
| 203811_s_at | DNAJB4 |
| 203973_s_at | CEBPD |
| 204472_at | GEM |
| 204621_s_at | NR4A2 |
| 204622_x_at | NR4A2 |
| 204748_at | PTGS2 |
| 205067_at | IL1B |
| 205249_at | EGR2 |
| 205409_at | FOSL2 |
| 206115_at | EGR3 |
| 206211_at | SELE |
| 206359_at | SOCS3 |
| 206360_s_at | SOCS3 |
| 207574_s_at | GADD45B |
| 207978_s_at | NR4A3 |
| 208078_s_at | SIK1 |
| 208960_s_at | KLF6 |
| 208961_s_at | KLF6 |
| 209189_at | FOS |
| 209304_x_at | GADD45B |
| 209305_s_at | GADD45B |
| 209582_s_at | CD200 |
| 209583_s_at | CD200 |
| 209774_x_at | CXCL2 |
| 209959_at | NR4A3 |
| 210056_at | RND1 |
| 210226_at | NR4A1 |
| 210764_s_at | CYR61 |
| 211143_x_at | NR4A1 |
| 211610_at | KLF6 |
| 212099_at | RHOB |
| 212665_at | TIPARP |
| 212724_at | RND3 |
| 213006_at | CEBPD |
| 213281_at | JUN |
| 213560_at | GADD45B |
| 213931_at | ID2 |
| 214056_at | MCL1 |
| 214057_at | MCL1 |
| 214105_at | SOCS3 |
| 215111_s_at | TSC22D1 |
| 215775_at | THBS1 |
| 215990_s_at | BCL6 |
| 216236_s_at | SLC2A14 |
| 216236_s_at | SLC2A3 |
| 216248_s_at | NR4A2 |
| 216979_at | NR4A3 |
| 218541_s_at | C8orf4 |
| 218880_at | FOSL2 |
| 218881_s_at | FOSL2 |
| 220266_s_at | KLF4 |
| 220890_s_at | APOLD1 |
| 221031_s_at | APOLD1 |
| 221841_s_at | KLF4 |
| 222088_s_at | SLC2A14 |
| 222088_s_at | SLC2A3 |
| 222162_s_at | ADAMTS1 |
| 37028_at | PPP1R15A |
| 39402_at | IL1B |
| 201169_s_at | BHLHE40 |
| 201170_s_at | BHLHE40 |
| 201695_s_at | PNP |
| 202627_s_at | SERPINE1 |
| 202628_s_at | SERPINE1 |
| 204121_at | GADD45G |
| 205266_at | LIF |
| 206432_at | HAS2 |
| 207844_at | IL13 |
| 208891_at | DUSP6 |
| 208892_s_at | DUSP6 |
| 208893_s_at | DUSP6 |
| 209101_at | CTGF |
| 210512_s_at | VEGFA |
| 210513_s_at | VEGFA |
| 211527_x_at | VEGFA |
| 212171_x_at | VEGFA |
| 216598_s_at | CCL2 |
| 220046_s_at | CCNL1 |

**Supplementary Table 5.** The Z-scores for menadione and gemcitabine in the prostate cancer cell lines.

|  | GSE21032 | | | | TCGA | | | |
| --- | --- | --- | --- | --- | --- | --- | --- | --- |
|  | PC3 | | VCaP | | PC3 | | VCaP | |
|  | Z-score | p-value | Z-score | p-value | Z-score | p-value | Z-score | p-value |
| MENADIONE | -2.39 | 0.016 | -8.69 | <0.001 | -0.58 | 0.552 | -12.56 | <0.001 |
| GEMCITABINE | -8.36 | 1.110 | -2.91 | 0.004 | -11.56 | <0.001 | -2.21 | 0.026 |
