# Supplementary figures and images for: Repurposing FDA approved drugs as radiosensitizers for treating hypoxic prostate cancer
Source: BMC Urol. 2021 Jul 1;21:96. doi: 10.1186/s12894-021-00856-x (PMC8247203; doi:10.1186/s12894-021-00856-x)

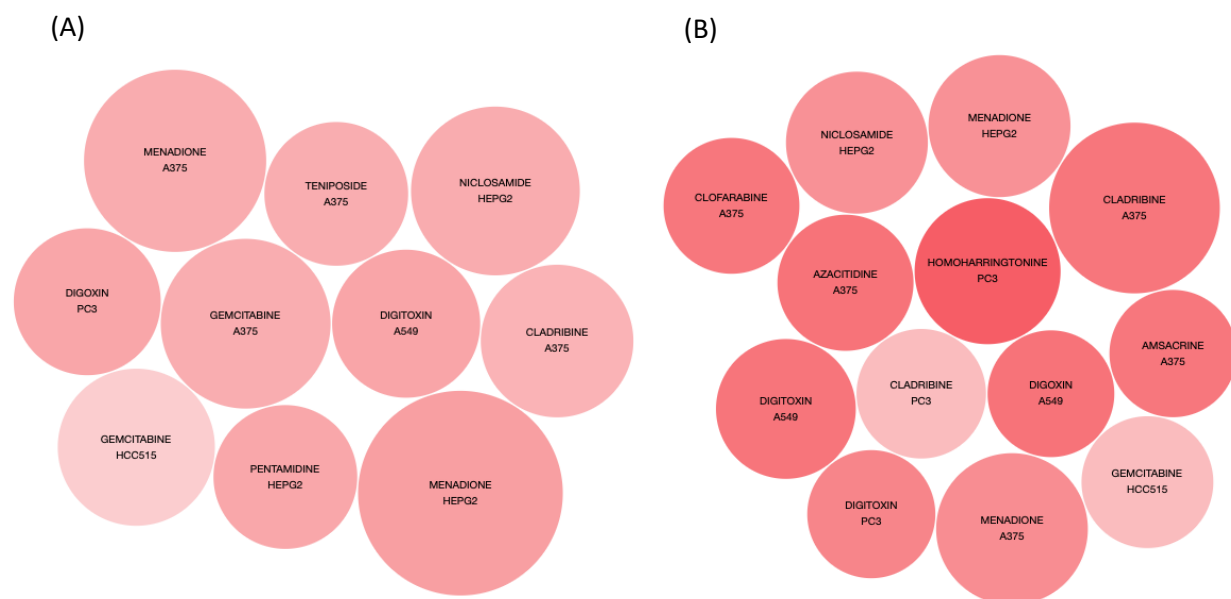

Supplementary Fig 1.

Supplement: Supplementary file 2 — Additional file 2. Supplementary Figure 1. Bubble plots representing the strength of the top negative connections identified by QUADrATiC. The larger the bubble the stronger the connection between the identified drug and the input genes. The drug and the cell line, that the connection was derived from, are shown in the bubbles. (A) GSE21032 (B) TCGA. [file 12894_2021_856_MOESM2_ESM.pdf]

(A)

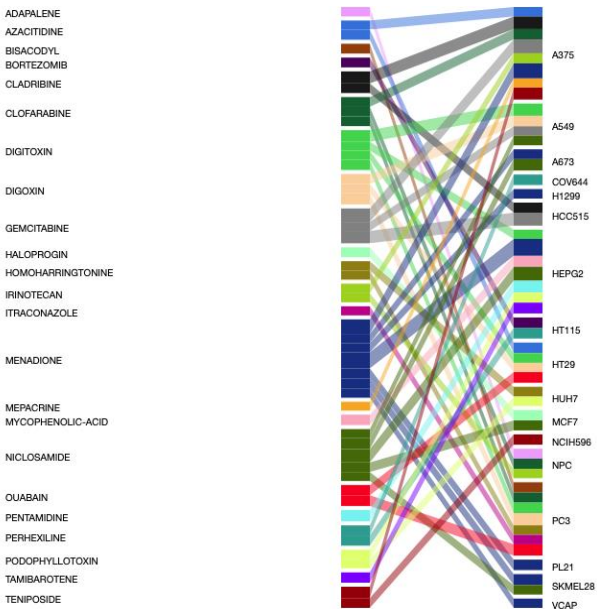

(B)

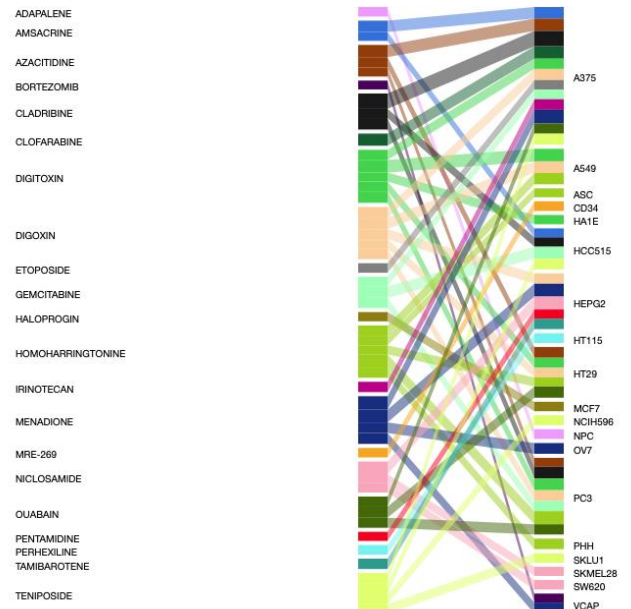

Supplementary Fig 2.

Supplement: Supplementary file 3 — Additional file 3. Supplementary Figure 2. Top drug and cell line connections identified by QUADrATiC. The plots represent the top ranked drugs and the cell line that the connection was derived from in the LINCs database. (A) GSE21032 (B) TCGA. [file 12894_2021_856_MOESM3_ESM.pdf]

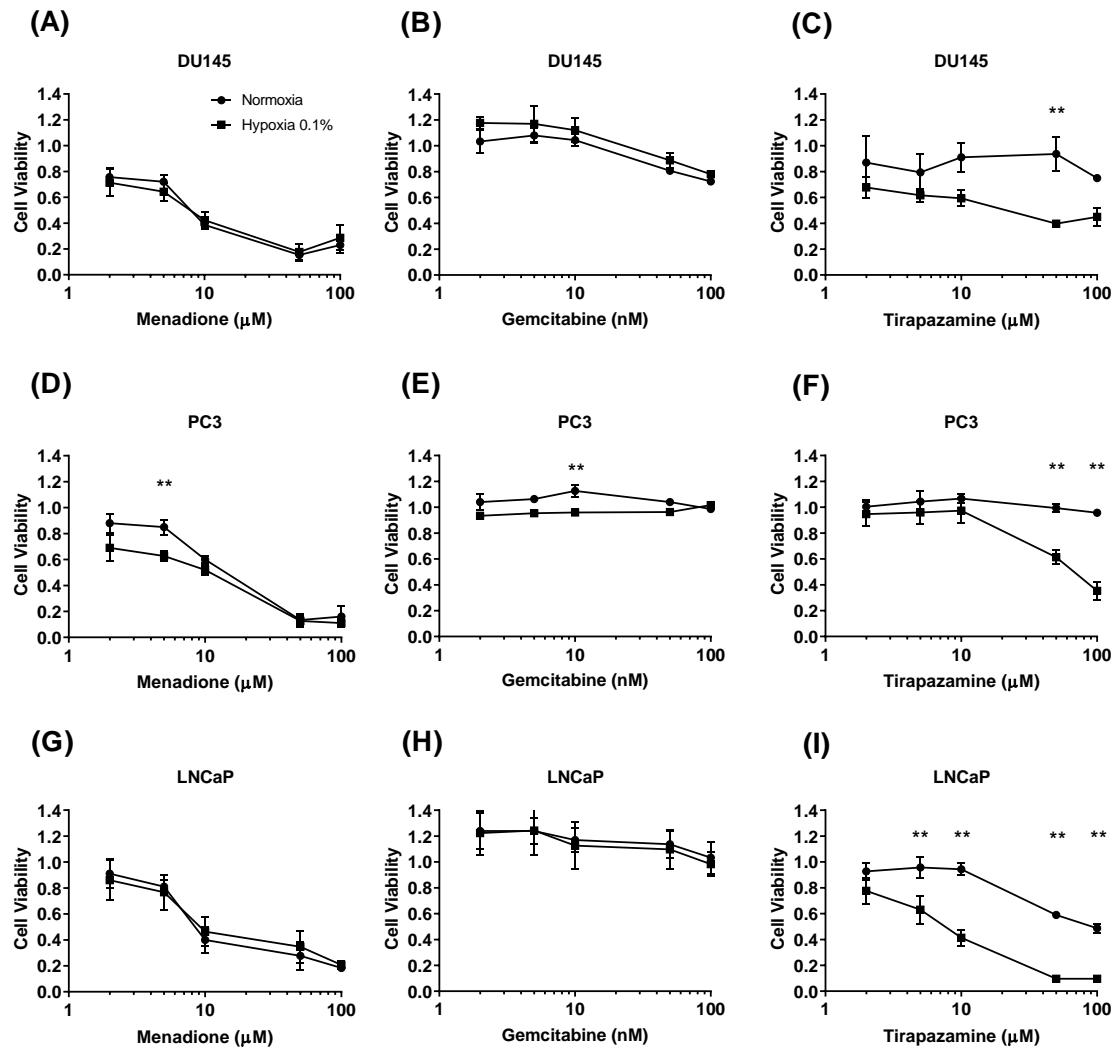

Supplementary Fig 3.

Supplement: Supplementary file 4 — Additional file 4. Supplementary Figure 3. No loss of cytotoxicity of menadione and gemcitabine in hypoxia versus normoxia after 24 h. DU145 (A–C), PC3 (D–F) and LNCaP (G–I) cell lines were exposed to menadione, gemcitabine or tirapazamine under normoxia or 0.1% O2 hypoxia. Three independent experiments were carried out, with six intra-assay replicates per experiment. Data points represent the mean ± SEM, statistical analysis was performed using a t-test with Holm-Sidak correction; **p < 0.01). [file 12894_2021_856_MOESM4_ESM.pdf]

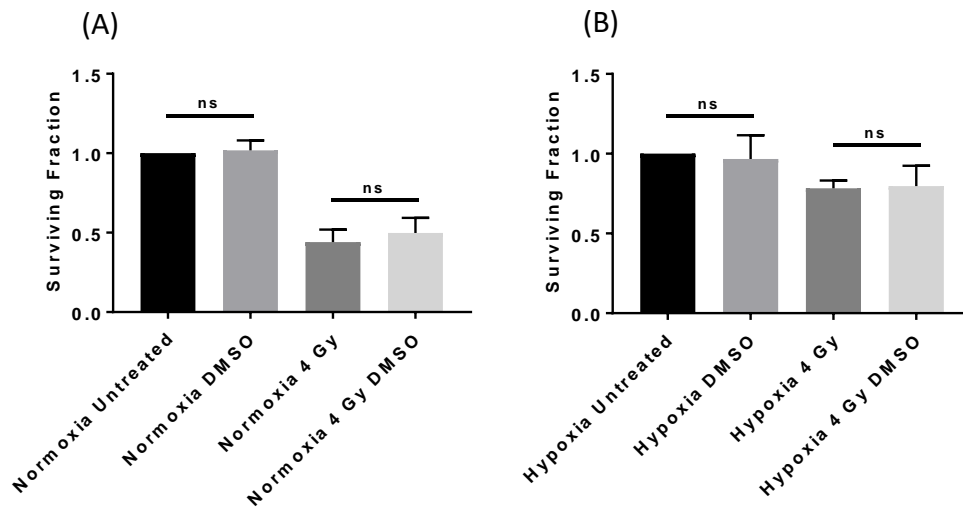

Supplementary Fig. 4

Supplement: Supplementary file 5 — Additional file 5. Supplementary Figure 4. (A) Under normoxia DMSO at a concentration of 0.02% (v/v) did not alter the surviving fraction of DU145 cells that were mockirradiated or irradiated with 4 Gy. (B) Under hypoxia DMSO at a concentration of 0.02% (v/v) did not alter the surviving fraction of DU145 cells that were mock irradiated or irradiated with 4 Gy. Data points represent the mean ± SEM of 3 biological repeats. [file 12894_2021_856_MOESM5_ESM.pdf]
